# Preoperative prognostic nutritional index and in-hospital mortality in neonates undergoing cardiac surgery for congenital heart disease: a single-center retrospective study

**DOI:** 10.3389/fnut.2026.1860048

**Published:** 2026-08-27

**Authors:** Heqi Zhang, Haoju Dong, Taibing Fan, Weijie Liang

**Affiliations:** Department of Children’s Heart Center, Central China Fuwai Hospital of Zhengzhou University, Zhengzhou, Henan, China

**Keywords:** cardiac surgery, congenital heart disease, in-hospital mortality, neonates, nutritional risk, prognostic nutritional index

## Abstract

**Background:**

Neonates undergoing cardiac surgery for congenital heart disease (CHD) represent one of the highest-risk subpopulations in pediatric cardiac surgery. Preoperative nutritional and immune reserve may substantially influence perioperative resilience, yet practical neonatal-specific nutritional risk markers remain insufficiently defined.

**Objective:**

To evaluate the association between preoperative prognostic nutritional index (PNI) and in-hospital mortality in neonates undergoing cardiac surgery for CHD, and to explore a neonatal-specific cutoff value for early risk stratification.

**Methods:**

This retrospective single-center cohort study included 433 neonates ( ≤ 28 days of postnatal age at surgery) who underwent primary cardiac surgery for CHD between January 2021 and December 2025. PNI was calculated from the final routine preoperative serum albumin (g/L) and total lymphocyte count ( × 10^9^/L) obtained before the index surgery. The optimal cutoff value was derived using receiver operating characteristic analysis and the Youden index. Associations between PNI and in-hospital mortality were assessed using nested multivariable logistic regression models and generalized additive models.

**Results:**

Overall in-hospital mortality was 3.70% (16/433), and mean PNI was 53.49 ± 13.05. Receiver operating characteristic analysis yielded an area under the curve of 0.661 (95% CI: 0.535–0.786; *p* = 0.020), with an optimal cutoff of PNI < 43.65, sensitivity of 56.3% (9/16), and specificity of 76.7% (320/417). Mortality was significantly higher in the low-PNI group than in the high-PNI group (9/106, 8.49% vs. 7/327, 2.14%; *p* = 0.007). In unadjusted analysis, low PNI was associated with higher in-hospital mortality (OR 3.85, 95% CI 1.08–14.29; *p* = 0.038). This association weakened after full adjustment (Model 3 OR 3.13, 95% CI 0.85–11.11; *p* = 0.086), likely reflecting limited event numbers and residual confounding.

**Conclusion:**

Lower preoperative PNI was associated with higher crude in-hospital mortality in neonates undergoing cardiac surgery for CHD. Because statistical independence was not retained in the fully adjusted model, PNI should be interpreted as an exploratory adjunctive screening marker rather than a stand-alone prognostic tool. Larger prospective multicenter studies are warranted to validate neonatal-specific thresholds and determine whether PNI-informed perioperative optimization can improve outcomes.

## 1 Introduction

Congenital heart disease (CHD) is the most common congenital anomaly, affecting approximately 8–10 per 1,000 live births, and remains the leading cause of congenital anomaly–related neonatal mortality worldwide (1, 2). Neonates—defined as infants within the first 28 days of life—with CHD represent a uniquely high-risk subgroup: their immature myocardium, limited physiological reserves, and the frequent need for complex neonatal cardiac surgery combine to generate substantially elevated perioperative morbidity and mortality rates compared with older pediatric patients (3–5). Despite significant advances in neonatal cardiac intensive care, in-hospital mortality after neonatal cardiac surgery remains between 3 and 10% at major centers (5, 6).

Nutritional vulnerability is a hallmark of the neonatal CHD population. Neonates have minimal caloric reserves, obligatorily high resting metabolic rates, and are critically dependent on continuous nutrient delivery for organ maturation, immune development, and myocardial growth (7–9). In the context of CHD, additional factors further compromise nutritional status: tachypnea and increased respiratory work reduce feeding tolerance; congestive heart failure and low cardiac output impair gastrointestinal perfusion and nutrient absorption; chronic or intermittent hypoxemia blunts appetite and increases anabolic requirements (7, 10). Preoperative protein–energy malnutrition in neonates undergoing cardiac surgery has been linked to heightened postoperative complications, including respiratory failure, infectious complications, impaired wound healing, and increased mortality (8, 11).

The Prognostic Nutritional Index (PNI), originally developed for adult gastrointestinal surgery risk assessment by Onodera and colleagues, is calculated as: PNI = serum albumin (g/L) + 5 × total lymphocyte count (× 10^9^/L). (12, 13) PNI has demonstrated prognostic relevance in adult oncological and cardiovascular surgical populations and, more recently, in mixed pediatric cardiac surgical cohorts (14–19). However, neonates have fundamentally different albumin distribution volumes, lymphocyte reference ranges, and immunological profiles compared with older children and adults, raising questions about the applicability of cutoff values derived from non-neonatal populations (7, 9, 10, 20, 21).

The only study specifically reporting PNI-mortality associations in neonates and infants undergoing CHD surgery is the cohort by Gu et al. (*n* = 3,082), which identified PNI as an independent predictor (19). However, neonates represented only a subset of that broader infant population, and neonatal-specific thresholds were not the central focus of that analysis. Similarly, Kaur et al. (*n* = 108) and Silva et al. examined PNI in pediatric cardiac surgery but did not concentrate exclusively on the neonatal age group (17, 18). Given the distinctive physiology, immunobiology, feeding vulnerability, and lesion severity encountered during the neonatal period, extrapolation from mixed pediatric cohorts may be inappropriate (4–7, 9, 10, 19–23). Accordingly, the present study aimed to evaluate PNI specifically in neonates undergoing cardiac surgery for CHD and to explore a neonatal-specific threshold for early risk stratification.

## 2 Materials and methods

### 2.1 Study design and population

**FIGURE 1:**
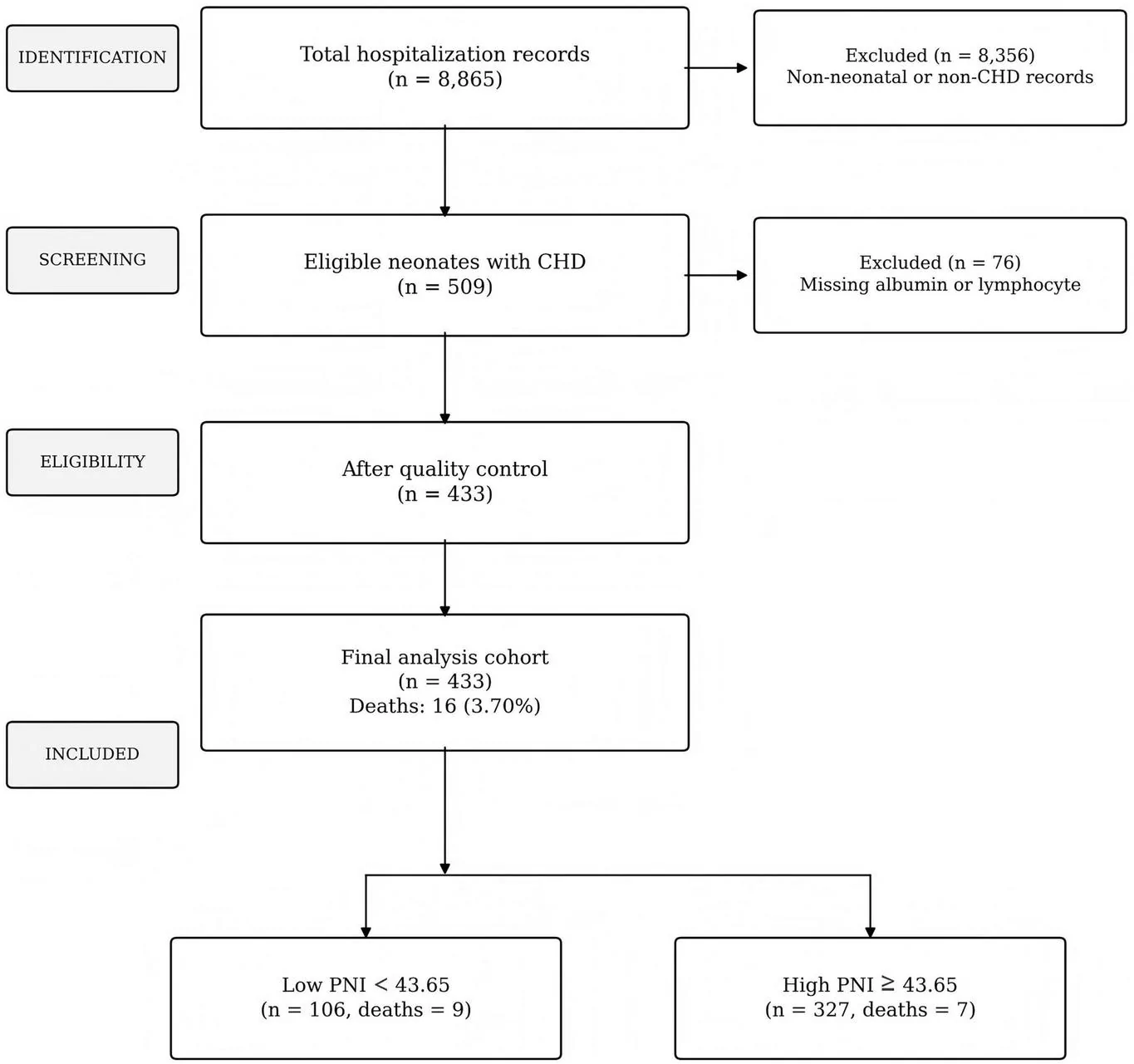
Patient enrollment flowchart for neonatal cohort.

This retrospective cohort study was conducted at Zhengzhou University Central China Fuwai Hospital. The study population comprised all neonates ( ≤ 28 days of postnatal age at surgery) who underwent primary cardiac surgery for CHD between January 2021 and December 2025. The final cohort comprised 433 neonates (Figure 1).

This study was approved by the Institutional Ethics Committee [Approval No.: (2025) Ke Lun Shen 18]. Individual informed consent was waived owing to the retrospective design.

### 2.2 Variable definition and PNI calculation

Preoperative variables included sex, body height (cm), body weight (kg), cyanotic CHD status, and laboratory parameters: serum albumin (g/L), total lymphocyte count ( × 10^9^/L), hemoglobin (g/L), fasting blood glucose (mmoL/L), and serum creatinine (μmoL/L). The serum albumin and total lymphocyte count values used for PNI calculation were extracted from the final routine preoperative laboratory assessment before the index cardiac surgery. PNI was calculated as: PNI = serum albumin (g/L) + 5 × total lymphocyte count ( × 10^9^/L).(12) Because albumin and lymphocyte counts vary across the neonatal period and with perioperative illness severity, no external adult or pediatric reference interval was used to define exposure groups. Instead, PNI was analyzed as a continuous cohort variable and by an institution-specific threshold derived from the present neonatal cohort. The primary outcome was in-hospital mortality.

### 2.3 Determination of the optimal PNI cutoff

The institution-specific PNI cutoff was independently determined by ROC curve analysis using the Youden index (J = sensitivity + specificity – 1). The resulting threshold was used to classify patients into low-PNI and high-PNI groups (24). This cutoff was selected to maximize the joint balance between sensitivity and specificity rather than to preferentially maximize either sensitivity or specificity. The numerical cutoff value and its diagnostic operating characteristics are reported in the Results section. The cutoff should be interpreted as an exploratory risk-stratification threshold rather than a stand-alone diagnostic rule.

### 2.4 Statistical analysis

Three nested multivariable logistic regression models were fitted: Model 1 (unadjusted), Model 2 (adjusted for sex, body weight, body height, cyanotic CHD), and Model 3 (Model 2 + hemoglobin, glucose, creatinine). Continuous variables in Table 1 were compared using Student’s *t*-test when approximately normally distributed and the Mann-Whitney U test otherwise; categorical variables were compared using the chi-square test or Fisher’s exact test, as appropriate. GAM with penalized spline smoothing was used to flexibly evaluate potential non-linearity between PNI and the log-odds of in-hospital mortality without imposing a strictly linear association. Missing values were imputed using missForest, a non-parametric random-forest-based iterative imputation procedure that handles mixed continuous and categorical variables and captures non-linear relations among variables (25). In the source database, PNI-related components were calculable for 8,746/8,865 records (98.66%); in the neonatal-age subset, they were calculable for 3,874/3,905 records (99.21%). R version 4.3 was used; *p* < 0.05 (two-tailed) was considered significant. For subgroup analyses, interaction was tested by adding a multiplicative PNI-by-subgroup term to the logistic regression model. For Figure 5, mortality proportions between PNI groups were compared using Fisher’s exact test because of the small number of deaths.

**TABLE 1:** Baseline characteristics stratified by PNI group (neonatal cohort).

Variable	Total (*n* = 433)	Low PNI < 43.65 (*n* = 106)	High PNI ≥ 43.65 (*n* = 327)	*P*-value
Male, n (%)	239 (55.2%)	61 (57.5%)	178 (54.4%)	0.654
Body height (cm)	47.89 ± 11.81	45.44 ± 10.85	48.68 ± 11.99	0.014
Body weight (kg)	2.69 ± 0.79	2.65 ± 0.61	2.71 ± 0.84	0.529
Cyanotic CHD, n (%)	172 (39.7%)	46 (43.4%)	126 (38.5%)	0.422
Serum albumin (g/L)	36.93 ± 6.49	31.21 ± 3.58	38.79 ± 6.13	< 0.001
Lymphocyte ( × 10^9^/L)	3.31 ± 2.05	1.74 ± 0.71	3.82 ± 2.08	< 0.001
Hemoglobin (g/L)	131.13 ± 30.01	136.80 ± 33.70	129.30 ± 28.47	0.025
Blood glucose (mmol/L)	5.24 ± 5.38	5.67 ± 2.75	5.10 ± 6.00	0.352
Serum creatinine (μmoL/L)	44.52 ± 23.41	53.26 ± 25.83	41.69 ± 21.83	< 0.001
PNI	53.49 ± 13.05	39.91 ± 3.17	57.89 ± 11.96	< 0.001
In-hospital mortality, n (%)	16 (3.70%)	9 (8.49%)	7 (2.14%)	0.007

CHD, congenital heart disease; PNI, prognostic nutritional index. Continuous: mean ± SD; categorical: n (%).

## 3 Results

### 3.1 Baseline patient characteristics

The final neonatal cohort comprised 433 patients (male: 239/433, 55.2%; mean body weight: 2.69 ± 0.79 kg). The overall in-hospital mortality rate was 3.70% (16/433). Mean PNI was 53.49 ± 13.05. The most frequent diagnoses were: ventricular septal defect (127/433, 29.3%), transposition of great arteries (63/433, 14.5%), patent ductus arteriosus (43/433, 9.9%), coarctation of aorta (42/433, 9.7%), and total anomalous pulmonary venous connection (34/433, 7.9%). Using the ROC-derived cutoff of 43.65, 106/433 patients (24.5%) were classified as low PNI (PNI < 43.65) and 327/433 (75.5%) as high PNI (PNI ≥ 43.65).

### 3.2 ROC analysis and optimal PNI cutoff

**FIGURE 2:**
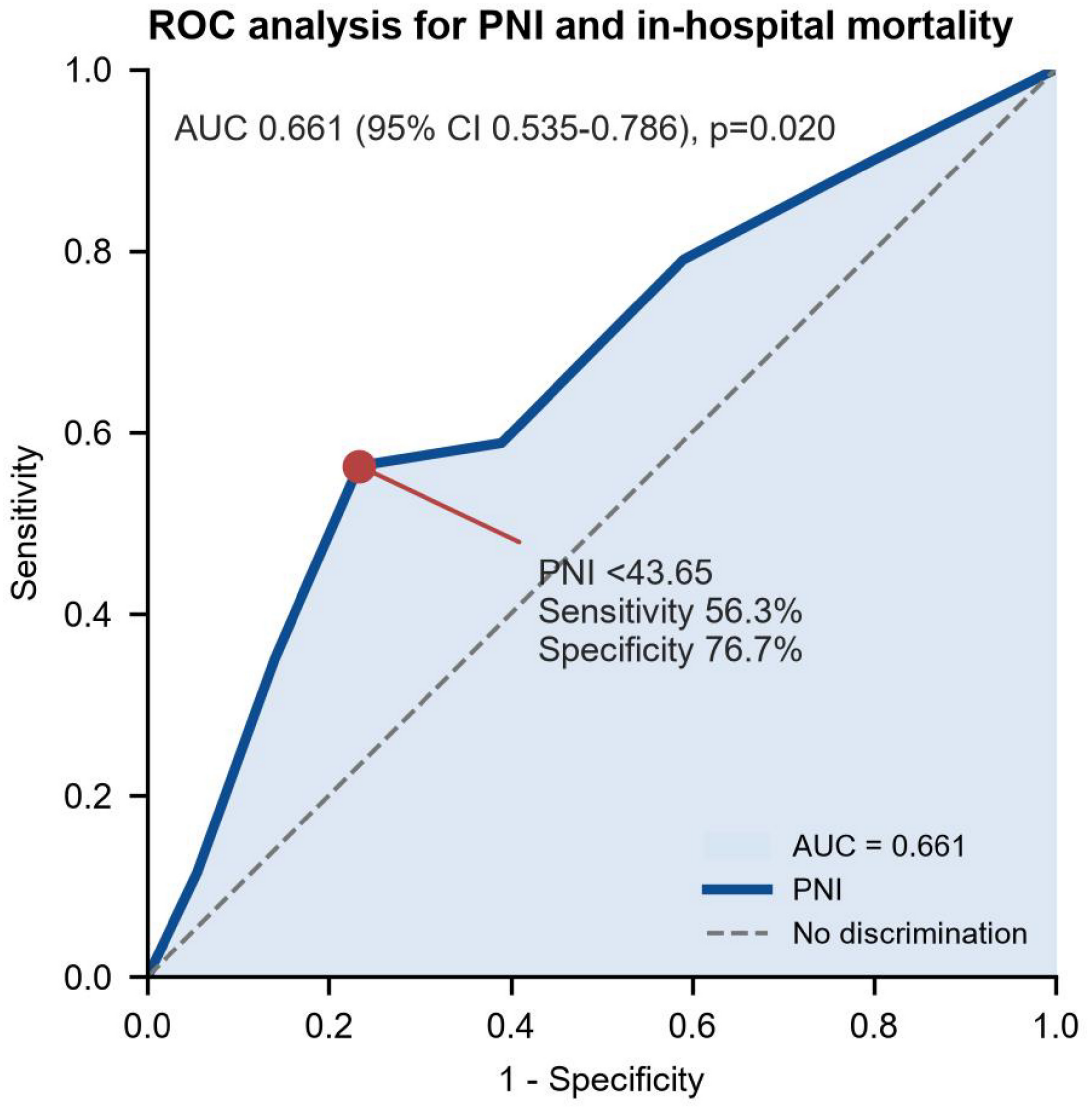
Receiver operating characteristic curve of PNI for predicting in-hospital mortality in neonates. The corrected ROC figure places the Youden-index point directly on the ROC curve. AUC = 0.661 (95% CI: 0.535–0.786; *p* = 0.020). The optimal cutoff was PNI < 43.65, with sensitivity 56.3% (9/16) and specificity 76.7% (320/417). Source data are provided as a grouped summary file.

ROC analysis demonstrated limited-to-fair discriminatory performance for in-hospital mortality in this neonatal cohort (AUC = 0.661; 95% CI: 0.535–0.786; *p* = 0.020). The optimal cutoff by the Youden index was PNI < 43.65, yielding sensitivity of 56.3% (9/16) and specificity of 76.7% (320/417) (Figure 2). The ROC threshold was used for exploratory risk stratification and did not identify a cutoff with both sensitivity and specificity exceeding 60% in this cohort.

### 3.3 Multivariable logistic regression analysis

Table 2 presents multivariable logistic regression results. To make the direction of risk easier to interpret clinically, odds ratios are presented per 1-SD decrease in PNI and for low PNI versus high PNI. In the unadjusted model, low PNI was significantly associated with higher in-hospital mortality (OR 3.85, 95% CI 1.08–14.29; *p* = 0.038). The association was directionally maintained after demographic adjustment (Model 2: OR 3.45, 95% CI 0.94–12.50; *p* = 0.062) but attenuated to non-significance in Model 3 (OR 3.13, 95% CI 0.85–11.11; *p* = 0.086). The wide confidence intervals reflect limited statistical power (16 deaths, events-per-variable ratio below 10).

**TABLE 2:** Multivariable logistic regression analysis (neonatal cohort).

Model	Variable	OR (95% CI)	*P*-value
Model 1 (unadjusted)	PNI per 1-SD decrease	1.25 (0.71, 2.22)	0.440
Model 1 (unadjusted)	Low PNI vs. High PNI	3.85 (1.08, 14.29)	0.038
Model 2 (+ demographics)	PNI per 1-SD decrease	1.12 (0.64, 2.00)	0.681
Model 2 (+ demographics)	Low PNI vs. High PNI	3.45 (0.94, 12.50)	0.062
Model 3 (fully adjusted)	PNI per 1-SD decrease	0.98 (0.54, 1.75)	0.937
Model 3 (fully adjusted)	Low PNI vs. High PNI	3.13 (0.85, 11.11)	0.086

Model 1: unadjusted. Model 2: adjusted for sex, body weight, body height, cyanotic CHD. Model 3: Model 2 + hemoglobin, glucose, creatinine.

### 3.4 Non-linear association

**FIGURE 3:**
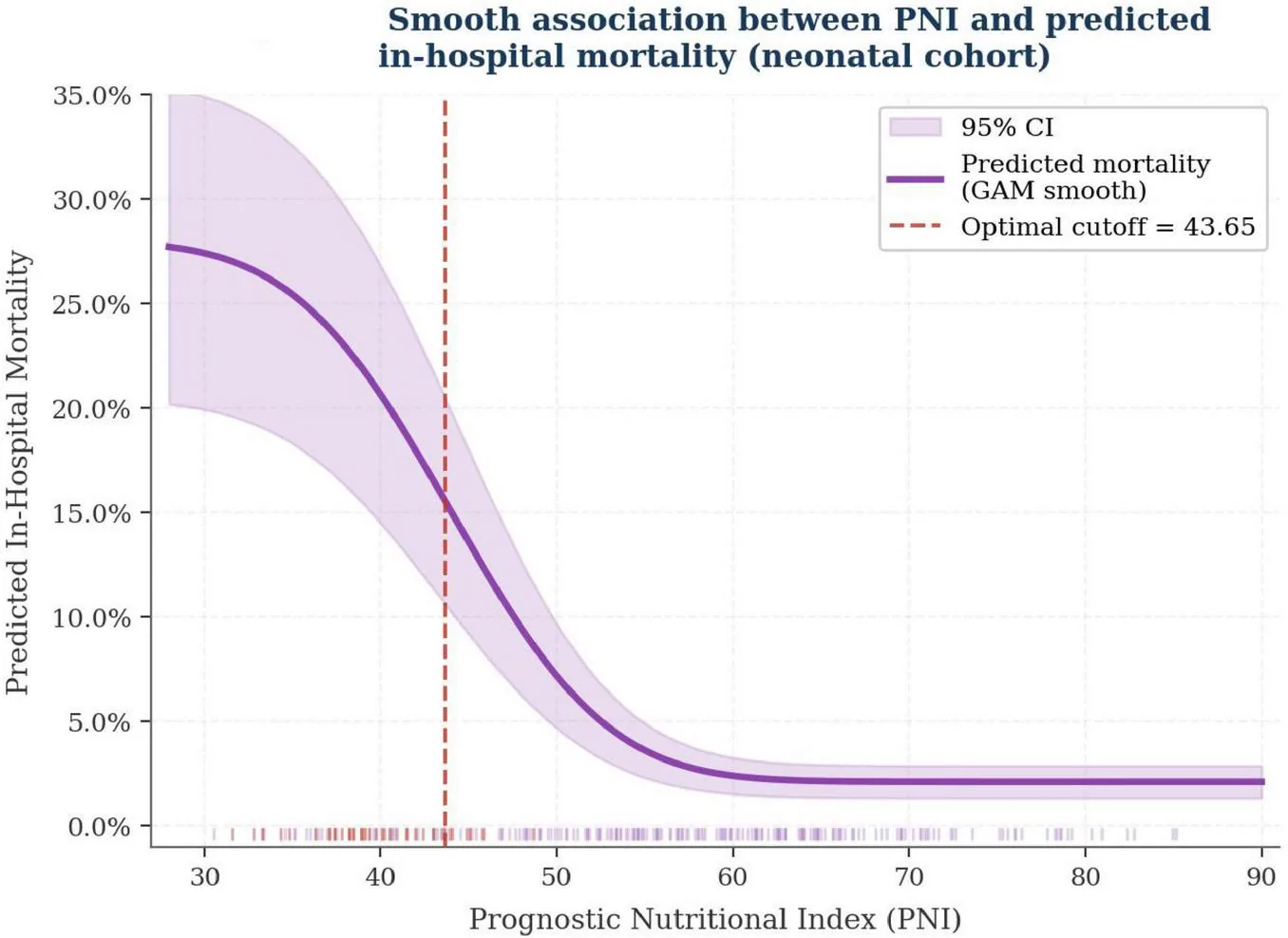
GAM smooth curve: association between PNI and in-hospital mortality in neonates. Penalized spline smoothing was used to assess potential non-linearity; the curve is exploratory because of the limited number of deaths.

**FIGURE 4:**
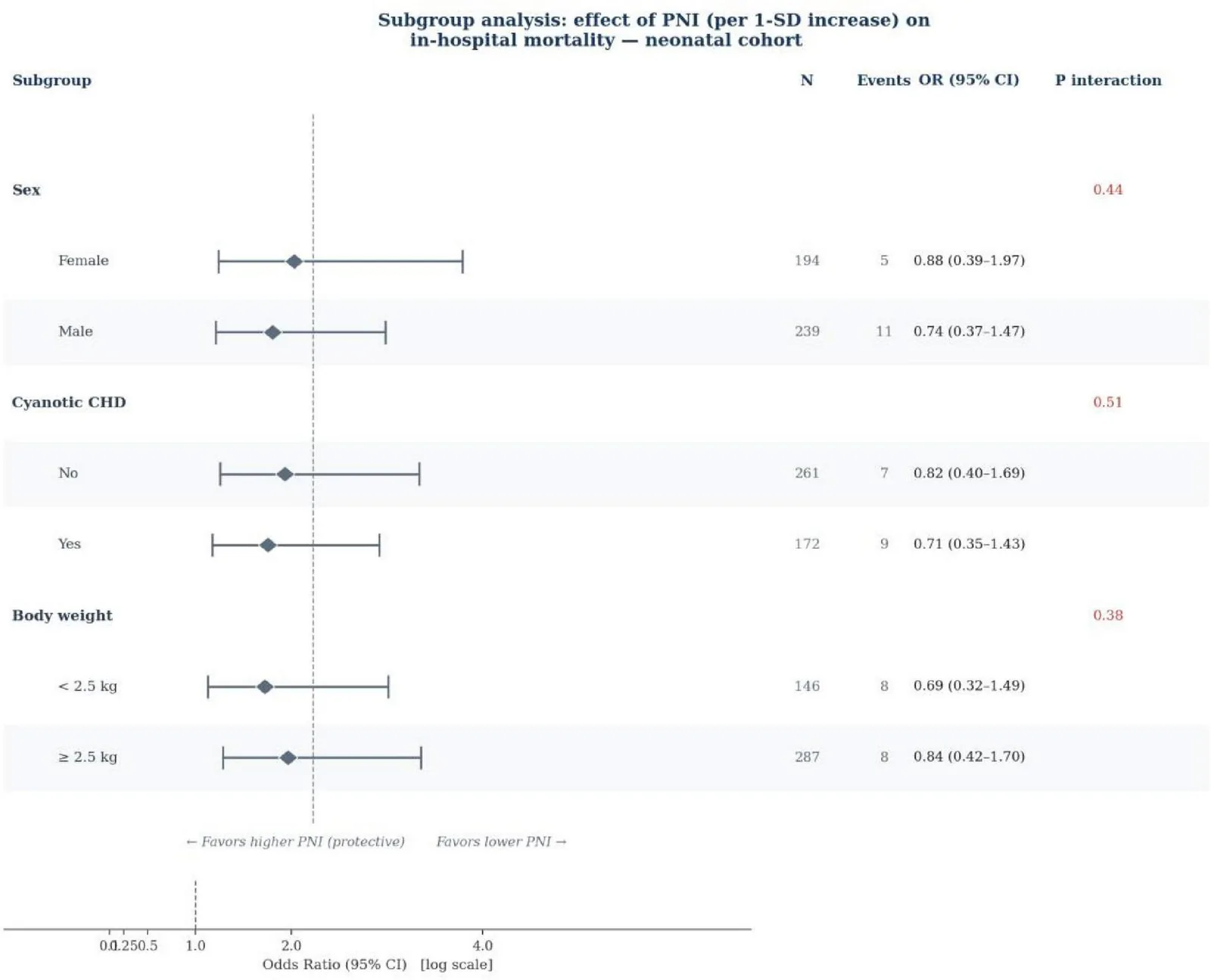
Subgroup forest plot: effect of PNI on in-hospital mortality in neonates. Interaction *p*-values were calculated by adding multiplicative PNI-by-subgroup interaction terms to logistic regression models.

**FIGURE 5:**
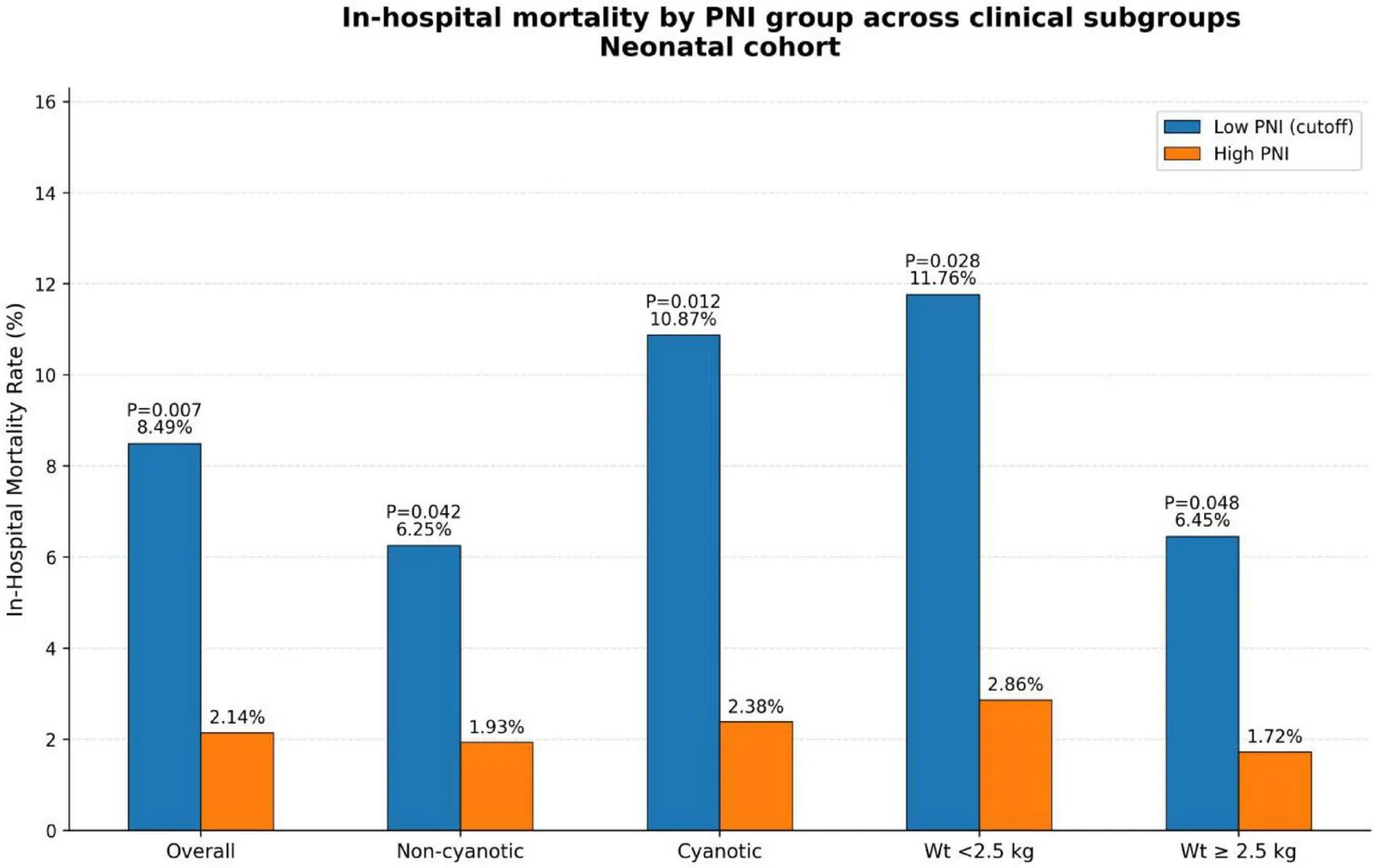
In-hospital mortality rates by PNI group in the neonatal cohort. Low PNI was defined as PNI < 43.65 and high PNI as PNI ≥ 43.65; between-group *p*-values were calculated using Fisher’s exact test.

GAM with penalized spline smoothing demonstrated an inverse trend between PNI and in-hospital mortality in the neonatal cohort, with wider confidence bands reflecting greater uncertainty (Figure 3). The steepest decline was observed between PNI values of approximately 30 and 45. We additionally performed an exploratory grouped internal calibration assessment using the reported low- and high-PNI strata. Model-predicted probabilities were anchored to the overall mortality rate and the fully adjusted low-versus-high PNI odds ratio, then compared with observed mortality in each stratum. Observed mortality was 2.14% (7/327) in the high-PNI group versus 8.49% (9/106) in the low-PNI group, while the corresponding model-anchored predicted probabilities were 2.66 and 6.89%, respectively. Because only 16 deaths occurred, this calibration assessment should be interpreted as descriptive and hypothesis-generating rather than a definitive validation of model calibration. Subgroup analyses showed no statistically significant interaction by sex, cyanotic CHD status, or body-weight category (all *P* for interaction > 0.05; Figure 4). Across the prespecified clinical subgroups, observed mortality was consistently higher in the low-PNI group than in the high-PNI group (Figure 5).

## 4 Discussion

### 4.1 Principal findings

In this dedicated neonatal CHD surgical cohort, lower preoperative PNI was associated with substantially higher crude in-hospital mortality. Patients classified into the low-PNI group had an almost fourfold higher mortality rate than those in the high-PNI group (8.49% vs. 2.14%), and the receiver operating characteristic-derived cutoff of 43.65 identified a clinically recognizable high-risk subgroup. Although statistical significance was attenuated after full adjustment, the direction of effect remained stable across all models, supporting the biological and clinical relevance of PNI in this vulnerable population (17–19, 26, 27).

### 4.2 Interpretation in the context of existing literature

Our findings are broadly consistent with the growing literature linking impaired nutritional and immune status to adverse outcomes after pediatric cardiac surgery (8, 11, 17–19, 26, 27). However, most prior studies evaluated mixed infant or pediatric cohorts rather than neonates specifically. That distinction matters. Neonates differ from older infants not only in body composition and metabolic reserve, but also in the developmental physiology of hepatic protein synthesis, immune-cell distribution, perioperative inflammatory response, and tolerance of catabolic stress (7, 9, 10, 20, 21, 23). The lower cutoff observed in the present study compared with mixed infant cohorts is therefore physiologically plausible and supports the concept that neonatal risk assessment should not simply import thresholds derived from older pediatric populations (19–21, 26).

The comparison with the recent cohort by Gu et al. is particularly informative. In that larger neonate-and-infant population, PNI showed independent prognostic value for mortality, but the model integrated a wider age spectrum and therefore reflected a broader biological range (19). By isolating neonates only, the present study addresses a clinically important gap and suggests that the threshold associated with elevated risk shifts downward in the immediate postnatal period. This observation aligns with the broader principle that biomarker interpretation in neonatal cardiac surgery should be age-calibrated rather than age-agnostic (4–6, 19–22).

### 4.3 Potential biological mechanisms

PNI integrates two biologically meaningful domains: visceral protein status, represented by serum albumin, and immune competence, represented by absolute lymphocyte count (12, 13, 20, 21, 28–31). In neonates with CHD, both components may be perturbed before surgery because poor feeding tolerance, increased work of breathing, low cardiac output, intestinal hypoperfusion or congestion, and systemic inflammation can reduce protein reserve and alter immune homeostasis (7, 9, 10, 23, 32, 33). A lower preoperative PNI may therefore reflect a state of combined metabolic fragility and impaired host defense rather than isolated undernutrition alone (11, 17–21, 26, 27).

This interpretation is clinically relevant because postoperative recovery after neonatal cardiac surgery depends on more than technical success in the operating room. Adequate protein reserve influences wound healing, capillary integrity, oncotic balance, and tolerance of cardiopulmonary bypass-associated inflammation, whereas immune competence may affect susceptibility to ventilator-associated complications, bloodstream infection, and delayed recovery (8, 11, 23, 27, 34). From this perspective, PNI may function less as a pure nutrition marker and more as an integrated index of physiological resilience (17–19, 26, 27, 35).

### 4.4 Clinical implications

The practical appeal of PNI lies in its simplicity. Both albumin and lymphocyte count are routinely available before surgery, making the index inexpensive, rapid, and easily generalizable in routine perioperative assessment (12, 13, 17–19, 26, 36). In routine practice, PNI could be calculated at the time of preoperative surgical evaluation, using the final routine laboratory assessment before the index operation, and reviewed during multidisciplinary surgical planning or preoperative intensive-care rounds. The present data do not support using PNI as a stand-alone determinant of surgical timing or candidacy. Instead, PNI may serve as an adjunctive screening tool to identify neonates who warrant closer nutritional assessment and perioperative surveillance.

A practical clinical algorithm can be proposed for implementation (Table 3). In brief, PNI should be calculated for neonates with CHD during preoperative surgical evaluation once serum albumin and lymphocyte count are available. The result should then be interpreted alongside postnatal age, growth trajectory, lesion complexity, cyanotic status, hemodynamic stability, renal function, and infection risk. For neonates with low PNI or rapid clinical deterioration, clinicians may initiate early dietitian involvement, individualized energy and protein targets, assessment of enteral feeding tolerance, and careful consideration of parenteral supplementation when enteral nutrition is insufficient or unsafe.

**TABLE 3:** Proposed clinical algorithm for integrating preoperative PNI into routine perioperative nutritional assessment in neonates with CHD.

Step	When and where	Suggested clinical use
1. Calculate	Preoperative surgical evaluation after routine laboratory results are available	Calculate PNI from serum albumin and total lymphocyte count obtained before the index operation to provide a rapid adjunctive marker of nutritional and immune vulnerability.
2. Interpret	Multidisciplinary surgical planning or preoperative intensive-care rounds	Interpret PNI together with age, growth trajectory, lesion complexity, cyanosis, hemodynamics, renal function, and infection risk, rather than as an isolated decision rule.
3. Optimize	Before surgery when the clinical condition allows	For low PNI, involve the nutrition team early, set individualized energy and protein targets, assess feeding tolerance, and consider supplemental nutrition if enteral intake is inadequate or unsafe.
4. Handover and monitor	Operating-room handover and postoperative intensive-care management	Communicate PNI category and the nutrition plan; tailor infection surveillance, fluid balance, renal monitoring, and nutrition advancement to overall risk.

This proposed pathway is intended to complement, rather than replace, established nutritional strategies for infants with CHD, including early multidisciplinary nutritional assessment, frequent growth monitoring, individualized enteral feeding plans, and escalation of nutritional support when oral or enteral intake is inadequate (36). Urgent or life-saving surgery should not be delayed solely because of a low PNI. Rather, a low value should prompt earlier recognition of nutritional and immunological vulnerability and a more explicit perioperative optimization plan.

PNI should therefore be interpreted within a multidimensional perioperative framework. Nutritional vulnerability in neonatal CHD is tightly intertwined with lesion complexity, preoperative instability, cyanosis, organ dysfunction, and the inflammatory burden of cardiopulmonary bypass (3–6, 11, 23, 26, 27, 34, 37–39). The greatest value of PNI may be in combination with clinical severity markers and procedure-related risk metrics, rather than as an isolated variable. Future work should examine whether PNI improves discrimination when added to established congenital heart surgery risk models and nomenclature-based risk stratification systems (38–40).

### 4.5 Statistical interpretation and reasons for attenuation after adjustment

The loss of formal statistical significance in the fully adjusted model should be interpreted cautiously rather than viewed as evidence against clinical relevance. Only 16 in-hospital deaths occurred in the present cohort, which constrained the events-per-variable ratio and limited multivariable model stability. Under these conditions, confidence intervals become wide and regression estimates are vulnerable to overadjustment and residual imprecision (19, 24, 25). Importantly, the effect estimate for high versus low PNI remained directionally protective after adjustment, suggesting that the main finding was weakened by limited power more than reversed by confounding (19, 26).

In addition, some adjustment variables may lie on pathways that are biologically related to nutritional vulnerability or overall disease severity, which can further attenuate observed associations. The present results should therefore be regarded as hypothesis-generating and clinically relevant mainly as a basis for prospective validation. This interpretation is supported by the crude risk separation between groups and by prior pediatric surgical studies of nutritional risk (8, 11, 17–19, 26, 27).

### 4.6 Strengths and limitations

This study has several strengths. It focused exclusively on neonates, used an institution-specific cutoff derived from receiver operating characteristic analysis, and evaluated the PNI-mortality relationship using both categorical and continuous approaches, including generalized additive modeling to explore non-linearity (19, 24, 25). These design features improve interpretability and help distinguish the present work from broader mixed-age pediatric analyses (17–19, 26, 27).

Several limitations should also be acknowledged. First, the retrospective single-center design introduces the possibility of selection bias and limits external generalizability. Second, the number of deaths was small, restricting statistical power and the complexity of multivariable adjustment. Third, detailed procedural risk indicators, such as surgical complexity scores, bypass duration, cross-clamp time, lactate dynamics, and preoperative hemodynamic support, were not incorporated into the current models, even though these variables are clinically relevant in congenital heart surgery risk assessment (4–6, 34, 37–40). Fourth, PNI was assessed at a single preoperative time point and therefore could not capture dynamic perioperative nutritional trajectories (14, 15, 23, 27). Finally, serum albumin may be influenced by inflammation, fluid status, hepatic function, and capillary leak, meaning that PNI should not be interpreted as a direct surrogate for malnutrition alone (12, 13, 17–19, 35).

### 4.7 Future directions

Future studies should prioritize multicenter validation of neonatal-specific PNI thresholds, external calibration across lesion subtypes, and integration with established congenital heart surgery risk stratification systems (38–40). It would also be valuable to determine whether serial perioperative PNI measurements improve prognostic performance over a single baseline assessment, as dynamic nutritional trajectories may carry additional information beyond one preoperative measurement (14, 15, 27). Most importantly, interventional studies are needed to test whether targeted optimization strategies in low-PNI neonates, such as intensified nutritional support, earlier enteral advancement when feasible, infection-prevention bundles, and multidisciplinary perioperative planning, can improve clinical outcomes.

## 5 Conclusion

In neonates undergoing cardiac surgery for CHD, a lower preoperative PNI was associated with higher crude in-hospital mortality, and the ROC-derived cutoff of 43.65 identified a subgroup with greater observed risk. Because the association was attenuated after full adjustment, these findings should be interpreted as exploratory. PNI may be considered as a simple adjunct to existing preoperative nutritional and clinical risk assessment, but it should not be used as an isolated decision rule. Prospective multicenter studies are needed to validate the threshold, define optimal timing for measurement, and test whether PNI-informed perioperative optimization improves outcomes (17–19, 23, 26, 27, 36).

## Data Availability

The raw data supporting the conclusions of this article will be made available by the authors, without undue reservation.
